# A new species of *Neossos* Malloch (Diptera: Heleomyzidae) from the Yukon Territory, Canada, and a revised key to the Nearctic species

**DOI:** 10.3897/BDJ.3.e6351

**Published:** 2015-10-05

**Authors:** Anna M. Solecki, Terry A. Wheeler

**Affiliations:** ‡ McGill University, Ste-Anne-de-Bellevue, Canada

**Keywords:** Taxonomy, acalyptrate, Beringia

## Abstract

**Background:**

The rarely collected genus *Neossos* Malloch contains three Nearctic and one western European species. Most known specimens have been collected from bird nests. Two specimens of an undescribed species of *Neossos* were collected by sweeping in subarctic tundra and a mesic meadow in the Yukon Territory, Canada. This represents a significant northward extension of the known Nearctic range of the genus.

**New information:**

*Neossos tombstonensis* **sp. n.** is described from the Yukon Territory. This represents the fourth described Nearctic species of *Neossos*. Although the type specimens were collected by sweeping, the species is predicted to be associated with bird nests, based on habits of other members of the genus. A revised key to the Nearctic species of *Neossos* is provided.

## Introduction

*Neossos* Malloch, 1927 is a rarely collected genus of acalyptrate Diptera primarily associated with bird nests, where the larvae are apparently saprophagous in nest material. Gilbert and Wheeler (2007) revised the Nearctic fauna of *Neossos* and recognized three species in the region: *Neossos marylandicus* Malloch, 1927 (associated with cavity-nesting passerine birds in eastern North America); *N. californicus* Melander, 1952 (associated with raptors in western United States and southern British Columbia); and *N. atlanticus* Gilbert & Wheeler, 2007 (associated with cliff-nesting seabirds in coastal eastern North America). In the course of a large-scale study of arthropod diversity and ecology in northern Canada, we collected two specimens of *Neossos* from the Yukon Territory, far north of the documented geographic range of Nearctic *Neossos* species. Those specimens are described here as *Neossos tombstonensis* **sp. n.**

## Materials and methods

Field-collected specimens were preserved in 95% ethanol and subsequently chemically dried using Hexamethyldisilazane. Type specimens are deposited in the Lyman Entomological Museum, McGill University, Ste-Anne-de-Bellevue, QC, Canada (LEMQ) and have been assigned unique specimen identifiers in the format LEM0000000. Genitalic dissection of the male holotype was made by detaching the posterior part of the abdomen, and heating it in 85% lactic acid on a heating plate for 10 minutes. Cleared genitalia were transferred to glycerin for examination and drawing, then stored in glycerin in a plastic microvial pinned with the specimen.

## Taxon treatments

### Neossos tombstonensis

Solecki & Wheeler, 2015 sp. n.

urn:lsid:zoobank.org:act:A7C54472-8048-461F-8C58-5DE8413622E4

#### Materials

- **Type status:** Holotype. **Occurrence:** recordedBy: NBP Field Party; sex: male; **Taxon:** class: Insecta; order: Diptera; family: Heleomyzidae; genus: Neossos; specificEpithet: tombstonensis; scientificNameAuthorship: Solecki & Wheeler, 2015; **Location:** continent: North America; country: Canada; stateProvince: Yukon Territory; verbatimLocality: Dempster Hwy nr North Fork Pass; verbatimElevation: 1200 m; decimalLatitude: 64.57942; decimalLongitude: -138.28212; geodeticDatum: WGS84; **Event:** samplingProtocol: sweeping; year: 2011; month: 6; day: 24; habitat: wet tundra; fieldNumber: wet replicate 3; **Record Level:** datasetID: LEM0110624; institutionCode: LEMQ
- **Type status:** Paratype. **Occurrence:** recordedBy: TA Wheeler; sex: female; **Taxon:** class: Insecta; order: Diptera; family: Heleomyzidae; genus: Neossos; specificEpithet: tombstonensis; scientificNameAuthorship: Solecki & Wheeler, 2015; **Location:** continent: North America; country: Canada; stateProvince: Yukon Territory; verbatimLocality: S Klondike Hwy, 18.2 km S Alaska Hwy, Robinson Road House; decimalLatitude: 60.44839; decimalLongitude: -134.84961; geodeticDatum: WGS84; **Event:** samplingProtocol: sweeping; year: 2012; month: 7; day: 9; habitat: mesic meadow; **Record Level:** datasetID: LEM0110625; institutionCode: LEMQ

#### Description

**Figure 1.**
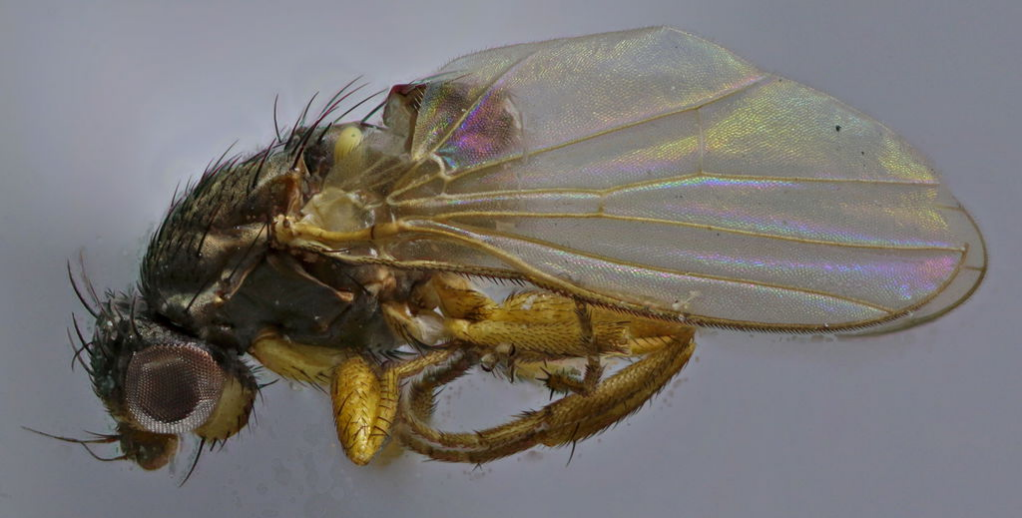
*Neossos tombstonensis*. Male holotype (abdomen dissected).

Generic characters as described by Gilbert and Wheeler (2007). Total length: 1.9 mm (♀) – 2.1 mm (♂). Frons slightly narrowing anteriorly, almost parallel-sided, yellow anteriorly, becoming darker at about 0.3 length of frons, black posteriorly; ocellar triangle microtomentose grey, subshining, shining black lateral to posterior ocelli and anterior to anterior ocellus; orbital plate same colour as ocellar triangle, more heavily microtomentose grey; face yellow, darker underneath antennae; clypeus brown, palpus and proboscis yellow; scape and pedicel yellow; first flagellomere yellow, browned dorsobasally on medial and lateral surface (darker in female); arista black-brown with slightly thicker yellow-brown base; postgena brown; gena dull yellow, more sclerotized, shining and brown at ventral margin (Fig. 1,) with four genal setae in addition to vibrissa and subvibrissal setae; strong seta posteroventrally on ventral margin of gena/postgena; genal height 0.4 times eye height; occiput same colour as ocellar triangle. Scutum same colour as ocellar triangle, uniform in colour and shading, heavily microtomentose; proepisternum, proepimeron, anepisternum, katepisternum, anepimeron, same colour as scutum (Fig. 1), except margins of sclerites paler, yellow-grey, katepimeron paler; scutellum same color as scutum. Wing length: 2.2 mm (♀) – 2.5 mm (♂). Legs yellow, coxae paler, distal tarsal segment, particularly of foreleg, slightly darker; hind tibia not noticeably expanded distally. Abdomen brown, sternites 2–5 same colour as tergites 2–5.

##### Male genitalia

**Figure 2.**
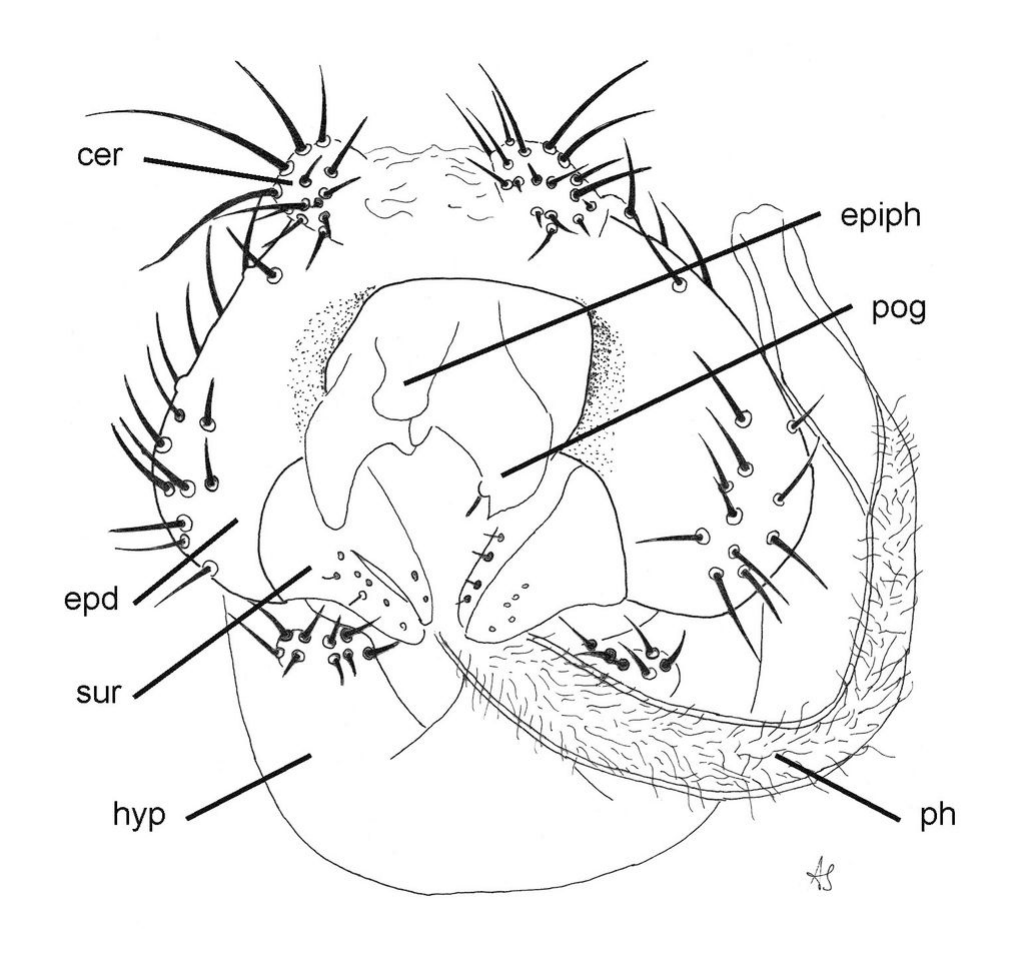
*Neossos tombstonensis*, male genitalia, ventral view. Abbreviations: cer - cercus; epd - epandrium; epiph - epiphallus; hyp - hypandrium; ph - distiphallus; pog - postgonite; sur - surstylus.

**Figure 3.**
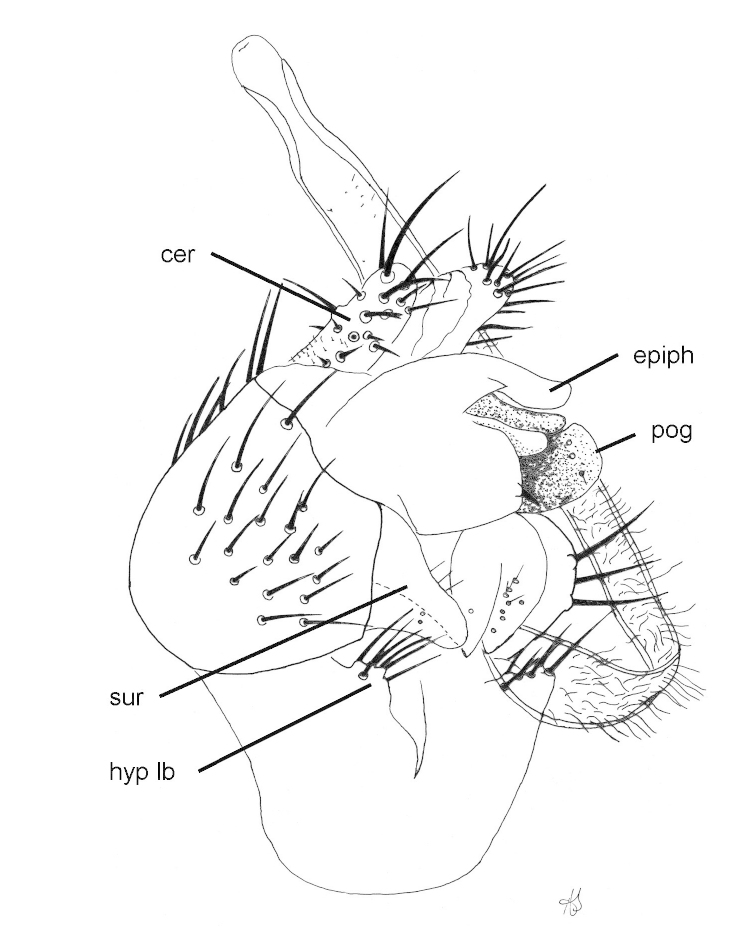
*Neossos tombstonensis*, male genitalia, ventrolateral view. Abbreviations: cer - cercus; epiph - epiphallus; hyp lb - hypandrial lobe; pog - postgonite; sur - surstylus.

Epandrium brown, rounded, wider than high; hypandrium pale brown, ventral hypandrial process with 9 setae; surstylus with outer lobe roughly triangular, narrowing distinctly in basal half; postgonite large; distiphallus with fine setulae for most of length except distally; epiphallus clavate (Figs 2, 3).

#### Etymology

The species is named for the Tombstone Mountains and Tombstone Territorial Park, where the holotype specimen was collected.

#### Distribution

Known only from the southern and central Yukon Territory, Canada.

## Identification Keys

### Key to the Nearctic species of *Neossos*

**Table:** 

1	Hind tibia expanded apically in anterior view, diameter at insertion of pre-apical dorsal seta at least 1.38 times diameter at midpoint of tibia and 3 times diameter at base (Fig. 4)	2
–	Hind tibia not obviously expanded apically, diameter at insertion of pre-apical dorsal seta 1.2 times diameter at midpoint of tibia, and less than twice diameter at base (Fig. 5)	3
2	Frons yellow-brown to brown posteriorly, never black; gena with 4–5 setae; median row of acrostichal setulae weak or absent; posterior and anterior thoracic spiracles the same size; outer lobe of surstylus narrowing sharply in apical third in lateral view. Western Nearctic (AZ, CA, UT, BC)	*Neossos californicus*
–	Frons dark-brown or black posteriorly; gena with 5–8 setae; median row of acrostichal setulae present; posterior thoracic spiracle larger than anterior spiracle; outer lobe of surstylus narrowing evenly in apical third in lateral view. Eastern Nearctic (QC)	*Neossos atlanticus*
3	Frons dark yellow posteriorly; proepimeron, proepisternum, anepisternum, katepisternum and anepimeron paler than scutum; surstylus narrowing evenly to apex in lateral view. Eastern Nearctic (MD, QC)	*Neossos marylandicus*
–	Frons black posteriorly; proepisternum, proepimeron, anepisternum, katepisternum, anepimeron, same colour as scutum; surstylus narrowing sharply in middle in lateral view. Northwestern Nearctic (YT)	*Neossos tombstonensis*

Although there are species-level differences in the shape of the male surstylus and epiphallus, the former is highly dependent on angle of view and the latter is subject to variation based on the preservation of the specimen and degree of collapse of the epiphallus. Both characters should be interpreted with caution.

## Discussion

Because they are apparently obligate associates of bird nests, specimens of *Neossos* are rarely collected. Of approximately 130 specimens studied by Gilbert and Wheeler (2007), all but one were collected or reared from bird nests or birds themselves. The single Palearctic species of *Neossos*, *N. nidicola* (Frey, 1930), known from Great Britain and Finland (Pape et al. 2015) is closely associated with bird nests. Although the collection of two specimens of *N. tombstonensis* by sweeping was surprising, the rarity of specimens was not. Multiple years of intensive sampling at the two localities from which types were collected, as well as other similar sites in the Yukon, have not produced another specimen.

The types of *N. tombstonensis* were collected in two distinct habitats. The North Fork Pass site is a wet tundra meadow north of treeline in the Ogilvie Mountain ranges. Dominant vegetation includes sphagnum mosses, grasses, sedges and ericaceous shrubs over a substrate with extensive permafrost. In contrast, the Robinson Road House site is a mesic meadow with a diverse assemblage of herbaceous plants dominated by Asteraceae, Fabaceae, and Poaceae, on a sand substrate in a clearing surrounded by spruce-pine-aspen forest.

Based on the known habits of the other described species of *Neossos*, it is likely that *N. tombstonensis* is also a nest associate. Each of the other three Nearctic species exploits a different microhabitat and host group: *Neossos marylandicus* in association with cavity-nesting passerines; *N. californicus* in raptor nests; and *N. atlanticus* in nests of cliff-nesting seabirds (Gilbert and Wheeler 2007). Several species of birds, including passerines and non-passerines, nest in both regions of the Yukon in which *N. tombstonensis* was collected, so it is impossible to speculate on the identity or nesting habits of the hosts.

## Supplementary Material

XML Treatment for Neossos tombstonensis

## Figures and Tables

**Figure 4.**
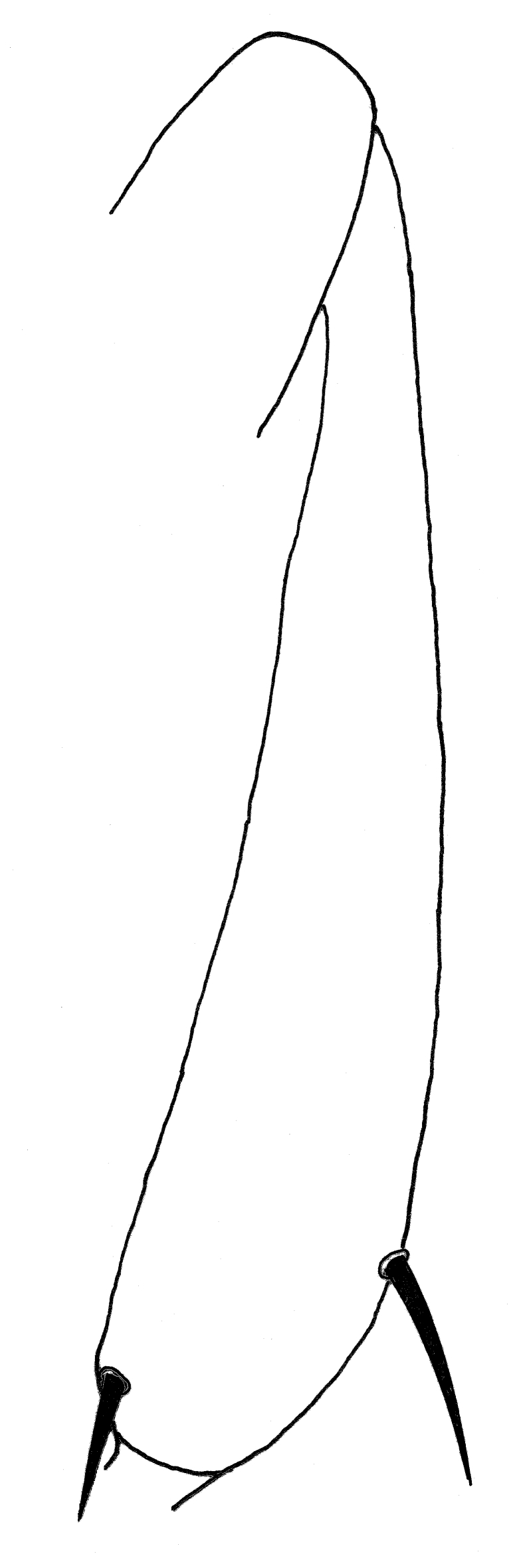
*Neossos atlanticus*, hind tibia, anterior view.

**Figure 5.**
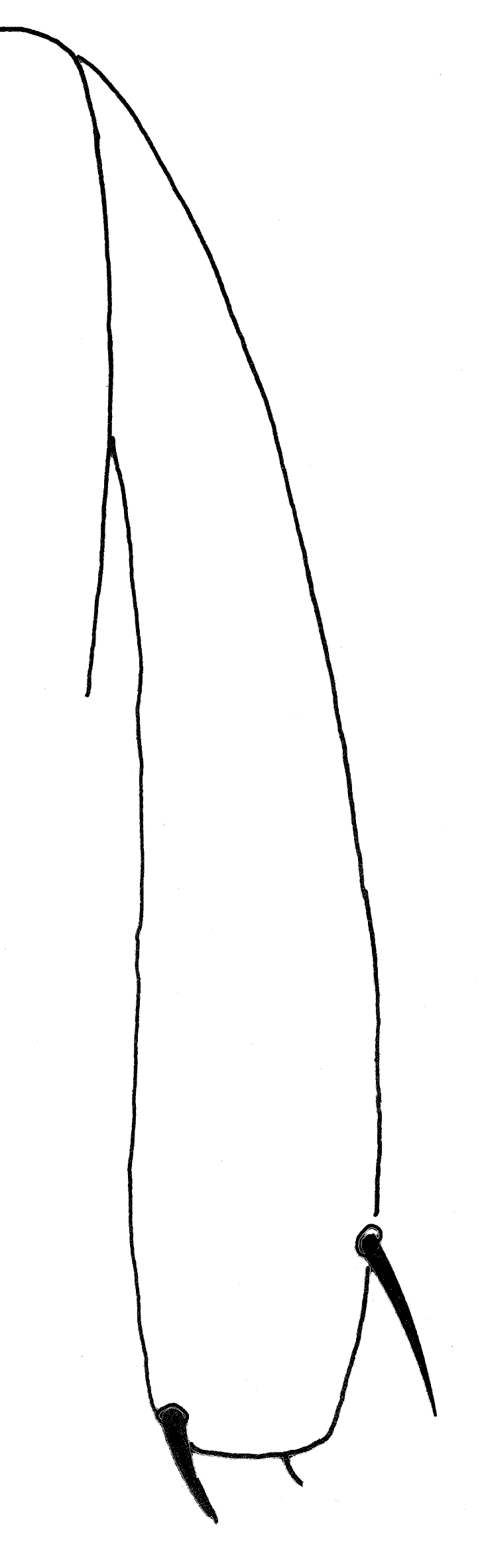
*Neossos marylandicus*, hind tibia, anterior view.
